# Structural Health Monitoring of a Prestressed Concrete Bridge Based on Statistical Pattern Recognition of Continuous Dynamic Measurements over 14 years

**DOI:** 10.3390/s18124117

**Published:** 2018-11-23

**Authors:** Wei-Hua Hu, De-Hui Tang, Jun Teng, Samir Said, Rolf. G. Rohrmann

**Affiliations:** 1 Harbin Institute of Technology (Shenzhen), Shenzhen 518055, China; huweihua@hit.edu.cn (W.-H.H.); tangdehui@stu.hit.edu.cn (D.-H.T.); 2 Federal Institute for Materials Research and Testing (BAM), 12205 Berlin, Germany; samir.said@bam.de; 3 Struktur Analyse & Bauwerks Monitoring (SABM) GbR, 10965 Berlin, Germany; rolf.rohrmann@gmx.de

**Keywords:** bridge, structural health monitoring, statistical pattern recognition, temperature effect

## Abstract

This work describes a vibration-based structural health monitoring of a prestressed-concrete box girder bridge on the A100 Highway in Berlin by applying statistical pattern recognition technique to a huge amount of data continuously collected by an integrated monitoring system during the period from 2000 to 2013. Firstly, the general condition and potential damage of the bridge is described. Then, the dynamic properties are extracted from 20 velocity sensors. Environmental variability captured by five thermal transducers and traffic intensity approximately estimated by strain measurements are also reported. Nonlinear influences of temperature on natural frequencies are observed. Subsequently, the measurements during the first year are used to build a baseline health index. The multiple linear regression (MLR) method is used to characterize the nonlinear relationship between natural frequencies and temperatures. The Euclidean distance of the residual errors is calculated to build a statistical health index. Finally, the indices extracted from the following years gradually deviate; which may indicate structural deterioration due to loss of prestress in the prestressed tendons.

## 1. Introduction

The vibration based structural health monitoring techniques are increasingly common. The basic premise is that structural responses, notably frequencies, modal damping and mode shapes, are functions of the physical properties of structure such as mass, stiffness and energy dissipation mechanisms. Therefore, changes in the physical properties will lead to detectable changes in the modal parameters. Applications of this technology are successful in both mechanical engineering and aerospace engineering. However, technological challenges are confronted in the application in civil engineering, considering the difficulty of recording the accumulated changes in a real structure over wide time scales under adverse environmental/operational conditions [1,2].

In the last few years, significant research efforts have been made to tackle the adverse environmental/operational effects on the measured structural response under the framework of statistical pattern recognition [3,4,5]. Rohrmann et al. observe that variations of frequencies in the Westend bridge caused by temperature may reach 10% according to continuous monitoring results from 1994 to 1997 [6]. Sohn et al. [7] use a linear filter model to remove temperature effects on daily variations of frequencies and build an internal confidence for potential structural change. Ni et al. [8] present that normal environmental change accounts for variation in the first ten modal frequencies from 0.20–1.52% based on monitoring of a 1177 m long cable-stayed bridge in 770 h, and both support vector machine and back-propagation neural network techniques [9] are implemented to accurately model the temperature effects on modal frequencies. Liu and DeWolf reported 6% annual variations of frequencies of a curved concrete bridge based on 932 events. A linear regression model and the resulting confidence interval were employed to treat the data in 2002 and 2005, and no significant structural change is found [10]. Gomez et al. investigated the variation of frequencies estimated from 1350 datasets and reported the approximately 5% continuous reduction from 2002 to 2010, which may indicate a gradual aging process. However, no temperature influences on the modal properties are considered in this case [11].

With the development of modern parametric system identification methods [12,13,14,15,16,17,18,19,20], more researchers focus on implementing a fully automated continuous dynamic monitoring system in order to not only detect possible simulated change but also understand the structural responses to environmental/operational variables. Magalhães et al. implemented an online dynamic monitoring system of an arch bridge and detect the simulated damage scenarios after removing the temperature effects using the principal component analysis (PCA) method and the multiple linear regression (MLR) approach [21,22]. Co-integration and a Kalman filter is proposed by Song et al. to eliminate the environmental effects on frequencies and further developed for on-line structure state evaluation [23,24,25]. Hu et al. analyzed the serviceability of a footbridge [26] on the basis of the massive amount of data recorded from continuous monitoring of these structures over several years. In [27], it was reported that the multiple linear regression (MLR) approach performs better than the Principal Component Analysis (PCA) method with regard to detecting the simulated damage by removing the environmental/operational effects on dynamic properties. Moser and Moaveni also implement a continuous dynamic monitoring system on a footbridge and attempt to remove the temperature effects using the MLR technique [28]. Cross et al. analyze the temperature, wind and traffic loads on the variations of frequencies on the basis of the long-term monitoring data from Tamar Suspension Bridge on the basis of response surface models [29]. In [30], Cunha et al. summarize the recent progress in the field of continuous dynamic testing and monitoring of bridges.

In particular, the Z24 Bridge is monitored during a nine-month period in the context of System Identification to Monitor Civil Engineering System (SIMCES) project, maximum difference ranging from 14% to 18% was observed for the first four natural frequencies; afterwards, the artificial damage was introduced to the bridge [31]. The Z24 Bridge has been a benchmark in the field of structural health monitoring and intensive research efforts were exerted on it. Peeters presented that the artificial progressive damage was successfully detected by applying autoregressive models with eXogenous inputs (ARX models) to remove environmental influences [31]. Kullaa used the control charts to detect the damage introduced on the bridge [32]. Yan stated that the local PCA method can efficiently eliminate the nonlinear environmental influences on dynamic properties [33]. Reynders employed the kernel PCA approach to remove the environmental effects in order to detect the damage [34]. Recently, Spiridonakos proposed the polynomial expansion technology for modeling the relationship between the frequencies and measured operational conditions; furthermore, the extracted features were used to detect the artificial damage exerted on the Z24 Bridge [35].

Alternatively, some researchers proposed different methods to tackle the environmental/operational effects. Instead of identifying modal parameters, the damage indicator is directly defined in time domain, according to the residual and its covariance matrix of block Hankel matrix. In a nominal, undamaged state, the damage indicator is quantified with the deviation of the structures. Afterwards, statistical hypothesis tests are carried out to judge whether new data can still be explained by the initial model [36]. Lin and Ren validate its efficiency in a rehabilitation process of a full-scale arch bridge under varying environments [37]. Döhler et al. employ both the modal parameters and the statistical null space-based damage detection methods to detect the artificial progressive damage of a prestressed concrete bridge. Unfortunately, no relevant environmental/operational changes are considered [38]. Recently, Tsogka et al. proposed the stretching method, mitigating the effects of environmental fluctuation and applied to long-term monitoring data of a historical monumental bell tower [39]. Wu et al. presents the rapidly convergent empirical mode decomposition (EMD) method for analyzing the environmental temperature effects on stay cable force [40].

Although extensive research has been conducted in the field of vibration-based structural health monitoring during the last two decades, most of the applications are still limited to model the environmental/operational effects or detection of the simulated damage. It is still rarely reported that realistic structural deterioration in a structure is captured by a vibration-based SHM system because the real deterioration has to accumulate over a long time-scale. While the monitoring histories of the above-described cases are less than ten years and thus no significant damage may have occurred during this period. Thus, verification of the ability to capture accumulated system change over a long time scale poses a fundamental challenge to the SHM technique. Meantime, another challenge is to validate that the detected change actually result from structures themselves instead of from the change of the sensing system over the long time scales. All of these challenges form obstacles for the vibration-based SHM technique to further transmit from a research topic to actual engineering practices [3].

The current research attempts to meet these challenges by implementing the statistical pattern recognition (SPR) technology for vibration-based SHM of an aging prestressed concrete highway bridge in order to track its deterioration process over 14 years from 2000 to 2013. Four steps of the SPR method are proposed in [3,4,5], consisting of (1) operational evaluation; (2) data acquisition and cleansing; (3) feature extraction and data compression and (4) statistical model development, are discussed on the basis of the continuous dynamic monitoring results. A clear “predefined” damage occurred in this bridge and a rehabilitation campaign was performed in 1990. Afterwards, the Department of Safety of Structures at the Federal Institute for Materials Research and Testing (BAM) was responsible for installing an integrated SHM system in 1994 and further update it several times from 1997 to 2000 [6,27]. From the year 2000 until now, over one hundred thousand data samples were recorded by the integrated monitoring system, consisting of measurements of velocity, strain and temperature under normal operational conditions. This paper focuses on removing the environmental/operational influences on the modal properties and presents the variation of the extracted statistical indicator. It begins with introduction of the Westend Bridge and the integrated monitoring system. Correlation analysis between environmental/operational variables and frequencies based on extensive measurements demonstrate that temperature is a main reason leading to change of frequencies. Afterwards, polynomial regression is used to remove the nonlinear temperature influences on frequencies in different orders and the corresponding residues are generated. Statistical health indicators are subsequently extracted by calculating the Euclidean distance of these residues. During the period from 2000 to 2013, a clear deviation of the statistical health indicators may suggest the loss of prestress in the prestressed tendons of the bridge, which can be also partially explained by numerical simulation of the inherent relation between decreasing tendencies of dynamic properties and the loss of prestress of the bridge.

## 2. Westend Bridge

**Figure 1:**
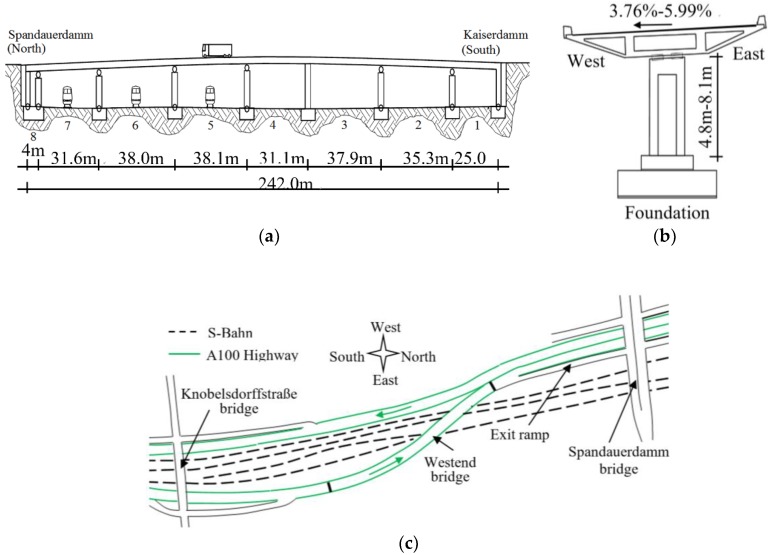
Westend Bridge on the A100 Highway in Berlin: (**a**) bridge elevation; (**b**) cross-section; (**c**) plan view.

**Figure 2:**
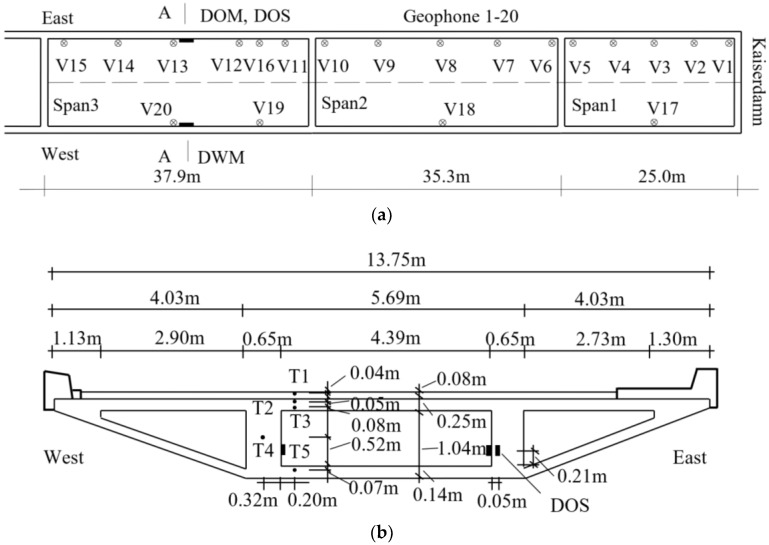
Deployment of velocity and temperature sensors: (**a**) plane view of Span 1–3 and locations of velocity sensors 1–20; (**b**) section A-A and position of temperature sensors T1–T5.

The Westend Bridge is a key structure of the National Highway A100 around Berlin, crossing the S-Bahn line and connecting the downtown core with the airport in the north. The structure, constructed in 1965, is a curved, prestressed-concrete box girder bridge. The entire length is 242 m with eight spans ranging from 5.0 m to 38.0 m. As shown in Figure 1a,b, the superstructure is supported by seven reinforced concrete (RC) columns with a hollow cylindrical cross-section. The height changes from 4.8 m to 8.1 m. The column in the middle of the bridge (span 3 and 4) is clamped at both ends in order to withstand horizontal loads. All the other columns are pin-ended and the two abutments at two sides of the bridge are formed by RC-cross walls. The whole bridge is built on foundation slabs. The superstructure is continuous and consists of three cell box girders with a maximum width of 13.75 m (Figure 2b). The travel direction of vehicles is from South to North and most of the heavy vehicles are on the east side (Figure 1c).

## 3. Integrated Health Monitoring System

In 1990, during the construction of drainage facilities for the three-cell box, two holes were made on the west side of the girder in order to support the drainage pipe. Unfortunately, three pre-stressing tendons crossing holes were accidentally cut. Thus, the fracture resistance of the whole structure was no longer guaranteed. Subsequently, the rehabilitation campaign was performed. Meanwhile, clear cracks are also observed in the conjunction part of the bridge slabs. As a result, the Federal Institute for Materials Research and Testing (BAM) is responsible for developing an integrated health monitoring system that continuously records the overall structural dynamic responses and local variations of the strains, crack and inclinations in the critical sections as well as the changes of the ambient factors under normal operational conditions [41,42]. The integrated health monitoring system is composed of a total of 32 sensors. In the current paper, the positions of the 20 vertical velocity sensors (V1–V20), five thermal transducers (T1–T5), four strain gauges (DOS, DWM and DOM) are clearly illustrated in Figure 2. Sixteen velocity sensors (V1–V16) are assigned along the east side of span 1 to 3 to measure the vertical responses because most of the heavy vehicles cross the bridge along this side. The rest of the 4 sensors (V17–V20) are arranged on the west side in order to identify the dynamic properties of the torsion modes. Likewise, a strain gauge is directly mounted on the surface of the main prestressed tendon on the east side (DOS) and two strain gauges are labelled on the surface of concrete webs in both west (DWM) and east (DOM) sides. As plotted in Figure 2b, 5 temperature sensors (T1–T5) are embedded in different positions in the section A-A with the initial purpose of investigating the influence of the thermal distribution on the variations of modal parameters. T1 is placed in the asphalt layer. Both T2–T3 and T5 are installed in the top and bottom flanges, respectively. T4 is put in the middle of the west web of the box girder [6].

The data acquisition system operates continuously. The sampling frequency is 128 Hz and the Butterworth filter is used. In time domain, every 32 s, 128 × 32 = 4096 points from each channel are acquired as a setup. The corresponding mean value, maximum value and root mean square values (RMS) of all measurements (velocity, temperature, strain, crack and inclination) are calculated. Every day, only the signals in time domain within one setup (32 s) are saved when the maximum velocity response in a reference channel occurs, and the rest of them are discarded due to limitation of storage space. In the period from March 2006 to July 2006 and from September 2007 to July 2008, the system stops due to loss of the power supply because of the construction of the new Spandauer Damm Bridge in the years 2006 to 2008. 

In frequency domain, two types of amplitude spectra are produced in two different time scales: one of them is 32 s in each setup; another one is every 50 successive setups in nearly 30 min. Regarding the bridge responses acquired from each channel, the fast fourier transform (FFT) algorithm is applied and an individual amplitude spectrum curve is computed on the basis of vibration signals consisting of 4096 points within 32 s. The rectangle window is used and the resolution is 128/4096 = 0.031 Hz. Every 50 successive setups in nearly 30 min, for each sensor, an averaged amplitude spectrum curve is obtained by averaging these curves produced in the 50 successive setups. The frequencies corresponding to peaks in the averaged amplitude spectrum curve are picked automatically and saved to storage devices. Only the last averaged amplitude spectrum curve in the midnight in a day is saved and the rest of the spectra are discarded, in order to save the storage space.

## 4. Continuous Dynamic Monitoring Results

The procedures of the traffic load and frequency estimations are introduced in the following sections. It should be emphasized that the continuous dynamic results reported in this section are in the time scale of one hour, on the basis of the analysis results in each setup with 32 s.

### 4.1. Estimation of Traffic Density and Its Fluctuation

Bridges are subjected to the dynamic forces imposed by moving vehicles, which generate additional dynamic effects on the bridges. In the design code, such effects are determined by the dynamic amplification factors (DAF). It is defined as the maximum total load effect (dynamic part and static part) divided by the maximum static load effect [43]. In order to investigate the effects of additional mass induced by traffic on the frequency estimates, the traffic density is approximately estimated by the static part of the strain recorded in the prestressed tendon (DOS). The dynamic part of the strain is discarded because it resulted from vehicle–bridge interactions.

**Figure 3:**
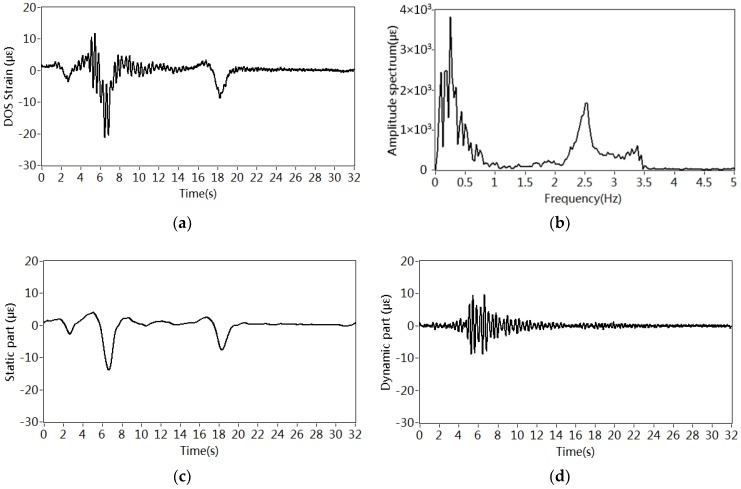

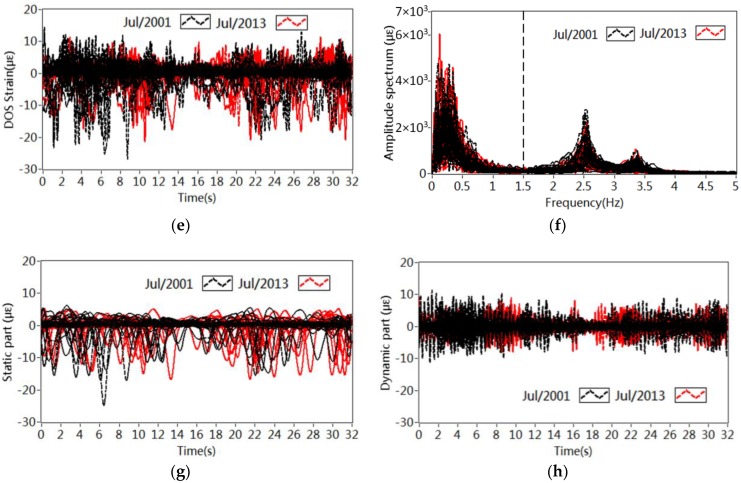
Estimation of traffic density: (**a**) Vibration signal recorded by DOS; (**b**) Frequency spectrum of signal in (**a**) in 5th July 2001; (**c**) Static part of signal in (**a**); (**d**) Dynamic part of signal in (**a**); (**e**) Vibration signal recorded by DOS; (**f**) Frequency spectra of signals in (**a**) in July 2001 and July 2013; (**g**) Static part of signals in (**e**); (**h**) Dynamic part of signals in (**e**).

Figure 3a shows a typical curve acquired by DOS within 32 s. Two significant drops indicate that heavy vehicles crossed the span 3. The amplitude spectrum of strain curve is plotted in Figure 3b. The frequency spectrum is separated into two parts by division frequency 1.5 Hz. One of them contains the spectrum below 1.5 Hz that represents the static effects of additional mass resulted from traffic. Another one is the spectrum above 1.5 Hz, which reflects the dynamic effects caused by vehicle–bridge interaction instead of additional mass. Two peaks around 2.5 Hz and 3.4 Hz are observed, which are identical to the first two bending modes that will be introduced in Section 4. Thus, the part of the spectrum above 1.5 Hz is discarded in the process of estimating traffic density.

Both spectra are transformed to the time domain again, as shown in Figure 3c,d. The graph in Figure 3c approximately reflects the quasi-static load of the vehicle while the Figure 3d depicts the structural vibration caused by the moving vehicles.

The feasibility of such a method is further validated by the prestressed tendon strain (DOS) measured in one month. Figure 3e plots the strain responses acquired in July of both 2001 and 2013. The corresponding amplitude spectrum curves are shown in Figure 3f. On one hand, many peaks are found around 2.5 Hz and 3.4 Hz, which reflect the contribution of dynamic part of strain due to the vehicle-induced bridge oscillations; on the other hand, most of the peaks are observed below 1.5 Hz and their amplitudes are rather higher than those around 2.5 Hz and 3.4 Hz. They approximately characterize the static part of the strain responses resulting from additional traffic mass. The corresponding static and dynamic responses in time domain are displayed in Figure 3g,h, respectively. Comparing these amplitudes in both static and dynamic parts, no clear differences are observed in July of both 2001 and 2013.

Subsequently, the root mean square (RMS) value of the static part of the strain recorded in the prestressed tendon (DOS), which is within a setup lasting 32 s, is calculated again in the period of one hour. Such an RMS value is used to appraise the traffic density crossing the bridge during one hour, which will be further used to investigate the effects of the frequency estimates within the same time scale, that is, in one hour.

**Figure 4:**
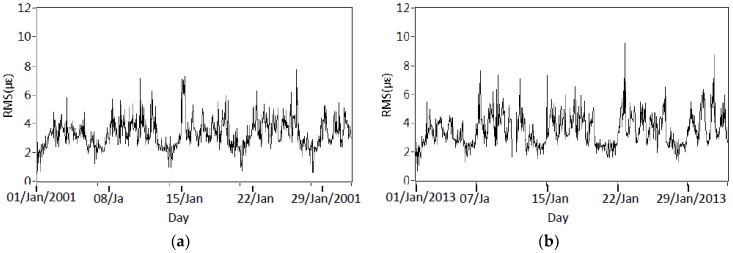

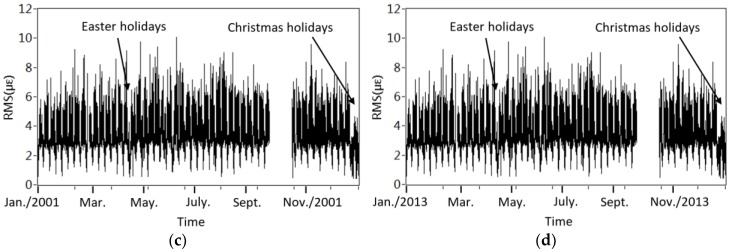
Variation of estimated traffic density: (**a**) Estimated traffic density in January 2001; (**b**) Estimated traffic density in January 2013; (**c**) Estimated traffic density in 2001; (**d**) Estimated traffic density in 2013.

Figure 4a,b shows the time history of the estimated traffic density in January during both 2001 and 2013. The clear daily fluctuation during working days is observed. Also, it is noted that the lower traffic density on the weekend is because heavy vehicles are not all allowed to cross the bridge. Figure 4c,d describes the annual variations of the estimated traffic density in both 2001 and 2013. Except for the weekly variations, lower traffic density is also found to occur in the Easter and Christmas holidays. Comparison of Figure 4a–d suggests that the RMS value of DOS in each hour is still at a similar level, which may suggest that there is no obvious change in traffic density in both 2001 and 2013.

### 4.2. Estimation of Natural Frequencies and Their Variations

The real-time integrated health monitoring system operates continuously. The basic data are dynamic signals acquired by every sensor with 128 Hz in 32 s. Regarding each velocity sensor, an amplitude spectrum is produced by applying FFT of the vibration signals acquired in every setup (4096 points) with a resolution 128/4096 = 0.031 Hz. In order to obtain smoother amplitude spectrum, multiple averages are performed on the basis of these spectra computed during each setup. The procedure consists of four steps: (1) Every 50 successive setups in nearly 30 min (32 s × 50/60 = 26.7 min ≈ 30 min). For each sensor, an averaged frequency spectrum can be calculated based on these frequency spectra produced in each individual setup; (2) Based on a 50 times averaged spectrum from one velocity sensor, the most excited peaks are automatically picked and the corresponding frequency values are selected; (3) On the basis of these most excited peaks selected from each velocity sensor during 50 successive setups, the corresponding frequency values from 16 velocity sensors are averaged again; (4) Finally, the most excited frequency values, during every half of an hour, are averaged again in order to estimate the frequency values of the bridge in the time scale of one hour, which are corresponding to the same scales used for temperature variation and traffic density estimation in Section 4.1 and Section 4.2.

**Figure 5:**
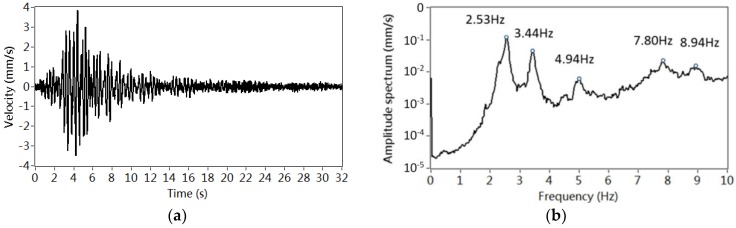
Identification of frequency: (**a**) Vibration signal in a setup from V16; (**b**) Frequency spectrum estimated by 50 measurements from sensor V16.

Figure 5a plots a typical vibration signal captured by V16 within 32 s and Figure 5b displays an averaged frequency spectrum on the basis of 50 measurements. The frequencies of the most frequently excited modes 1 to 5 are around 2.5 Hz, 3.4 Hz, 4.9 Hz, 7.8 Hz and 8.9 Hz, respectively.

In [44], the results of experimental modal test were introduced. Meanwhile, a finite element model was developed and calibrated by the experimental results [45]. The three-cell concrete box girder is modeled by the combination of beam elements and shell elements. Two ending walls are simulated by shell element while the Euler beam element used to model the reinforced columns. The top of the column is hinged with the girder while the bottom of the column is simulated with spring element to account for the elasticity of soil. The prestress of reinforcing tendons are considered by raising the elastic modules of the shell elements.

In the model updating procedure, the material parameters such as young’s modulus, shear modulus, mass density, mass moments of inertia and the local design parameters like cross section areas torsional constants, plate thicknesses and spring stiffness are taken into considering. The modal parameters should correspond to the experimental ones within the following limits: eigen-frequency deviation does not exceed ±0.3 Hz and corresponding MAC value is larger than 90%.

**Figure 6:**
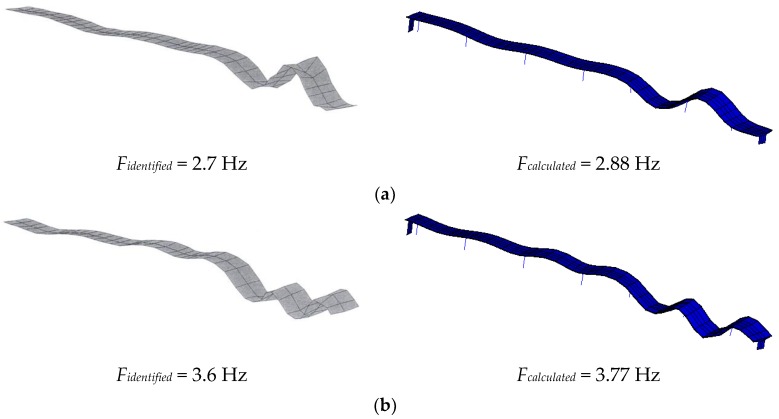

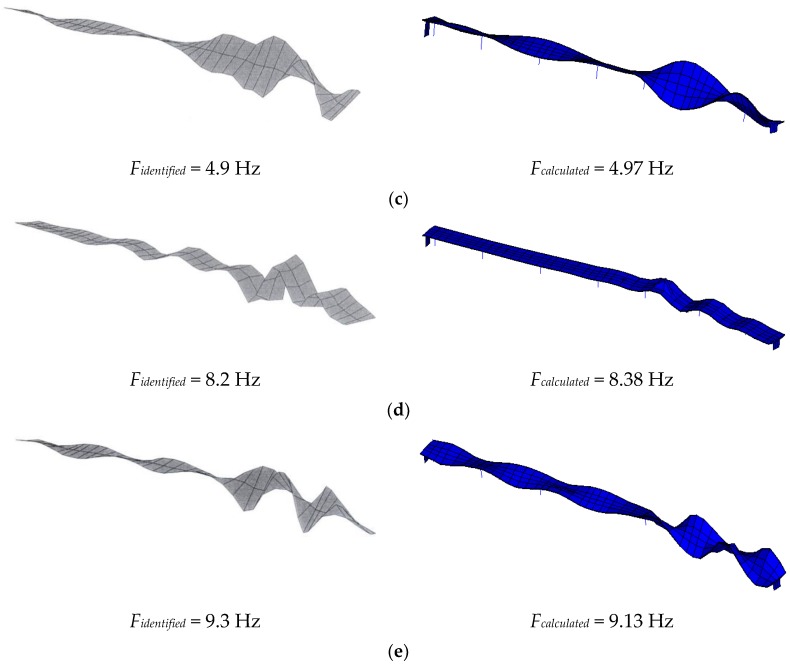
Mode shapes identified by modal test (left) and calculated by finite element analysis (right), corresponding to the most excited modes 1 to 5: (**a**) 1st mode; (**b**) 2nd mode; (**c**) 3rd mode; (**d**) 4th mode; (**e**) 5th mode.

The updated mode shape and frequency of most excited five modes are shown in Figure 6.

**Figure 7:**
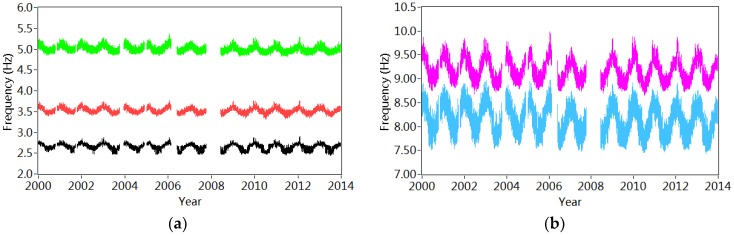
Variation of the estimated frequencies from 1st January 2000 to 31st December 2013: (**a**) 1st, 2nd and 3rd mode; (**b**) 4th and 5th mode.

The structural natural frequencies are estimated by averaging the identified frequencies from different sensors. Finally, the mean frequencies from two 50 successive setups are calculated again to generate the natural frequencies on the basis of the dynamic responses measured in one hour. Figure 7 shows the variations of the frequencies in five modes from 2000 to 2013. The obvious annual fluctuation is observed due to the environmental/operational influences. Meanwhile, the frequencies in higher modes are more sensitive with these environmental/operational variations. 

Table 1 and Table 2 list the statistical results of the annual variations of the frequencies corresponding to both bending and torsional modes. It is observed that the annual relative variations change from 7.4% to 17.5%, which shows further evidence the significant environmental/operational effects. It is also interesting to note that the annual averaged frequencies in different modes decrease slightly after 2006–2008, when a new bridge was constructed nearby. It may indicate the occurrence of the structural modifications that will be further explained in the following sections.

## 5. Environmental/Operational Effects on Variations of Frequencies

### 5.1. Effect of the Traffic Density on the Estimated Frequencies

**Figure 8:**
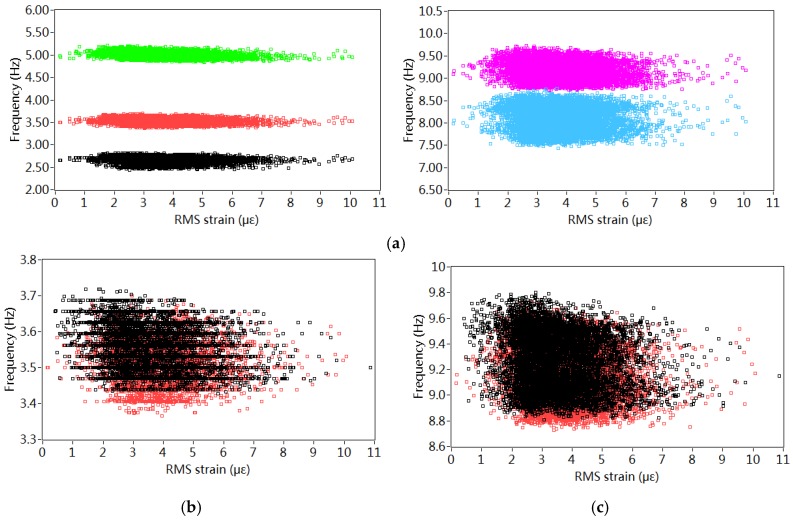
Effect of traffic density on the estimated frequencies: (**a**) Effect of traffic density on the frequencies in 2013; (**b**) Effect of traffic density on the frequencies in different years (3.4 Hz); (**c**) Effect of traffic density on the frequencies in different years (8.9 Hz).

The traffic density crossing the bridge is approximately evaluated by the RMS values of the static part of the measured strain DOS in one hour. The influence of the traffic load on the variation of frequencies is illustrated in Figure 8. Figure 8a shows the relationships between the estimated traffic density with the identified frequencies in 2013. No obvious influences of the traffic density are observed on the frequencies in different modes. Figure 8b,c compares the effect of the estimated traffic density on the identified frequencies around 3.4 Hz and 8.9 Hz in both 2001 and 2013. It is also noted that no clear influence from the traffic density is observed. However, it is interesting to notice that the frequencies in two modes around both 3.4 Hz and 8.9 Hz in 2013 drop slightly compared to those identified in 2001.

### 5.2. Effect of the Temperature Records on the Estimated Frequencies

**Figure 9:**
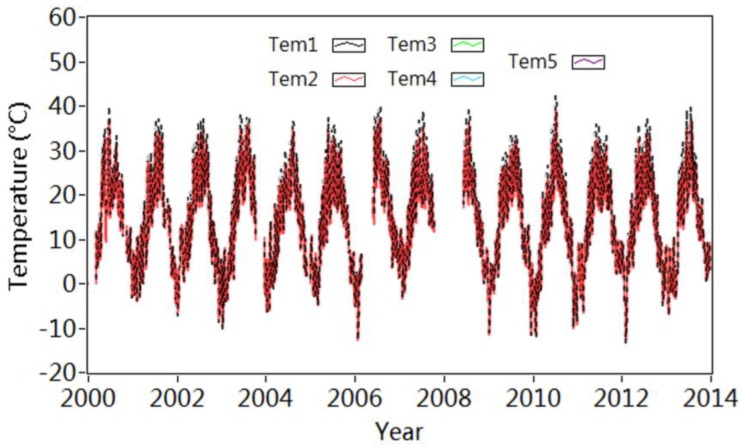
Variation of temperature acquired from T1 to T5.

The mean values of temperature during every 32 s are continuously recorded. During one hour, these mean values calculated based on every setup are averaged again for further analysis. Figure 9 provides the time history of temperature recorded by T1–T5 from 2000 to 2013. Both the highest and lowest temperatures are captured by T1 in the asphalt layer. The former one is 49.3 °C recorded in July 2010 and the latter one is −14.6 °C measured in February 2012.

**Figure 10:**
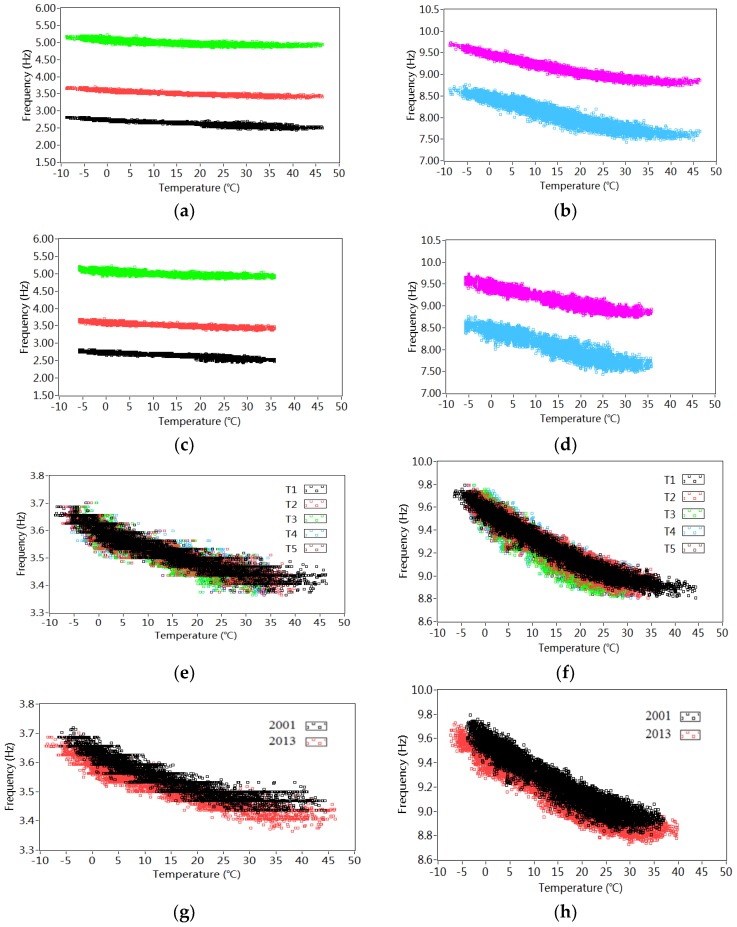
Effect of temperature on the estimated frequencies: (**a**) Effect of temperature recorded by T1on the identified frequencies in 2013; (**b**) Effect of temperature recorded by T1 on the identified frequencies in 2013; (**c**) Effect of temperature recorded by T4 on the identified frequencies in 2013; (**d**) Effect of temperature recorded by T4 on the identified frequencies in 2013; (**e**) Effect of temperature recorded indifferent positions on the frequencies (3.4 Hz); (**f**) Effect of temperature recorded indifferent positions on the frequencies (8.9 Hz); (**g**) Effect of temperature of frequencies in different years (3.4 Hz); (**h**) Effect of temperature of frequencies in different years (8.9 Hz).

Figure 10 describes the effect of temperature on the frequencies. In 2013, the thermal sensor T1 installed in the asphalt changes from −8.8 °C to 46.3 °C while the T4 in the web concrete falls in the range from −5.7 °C to 35.7 °C. It can be seen from Figure 10a,b that the temperature recorded by both T1 and T4 in 2013 have nonlinear influences on the variation of frequencies in all 5 modes. The frequencies in different modes decrease with the temperature increase. Figure 10c,d plots the relationship between the identified frequencies around 3.4 Hz as well as 8.9 Hz and the temperature measured by different sensors T1–T5. Regarding these two modes, similar nonlinear influences from different temperature records on the identified frequencies are observed. Figure 10e,f compares the effects of temperature measured by T1 in both 2001 and 2013 on the frequencies around 3.4 Hz and 8.9 Hz. It is noted that the temperature records T1 have nearly the same influence on the estimated frequencies of both modes. As shown in Figure 10g,h, the frequencies in both modes in 2013 decrease slightly compared to those in 2001, as discussed in Table 1 and Table 2 and Figure 9, indicating possible structural modifications.

In the following sections, the nonlinear influences of the temperature records on the identified frequencies will be removed and the structural modifications will be further demonstrated.

## 6. Elimination of Environmental Effects and Detection of Structural Modifications

### 6.1. Proposed Methodology

The basic assumption of the vibration-based health monitoring method is that structural modifications will alter the stiffness, mass or energy dissipation properties of a system, which will further change the structural responses. However, with regard to an infrastructure system under operational conditions, the structural responses are strongly dependent on the environmental/operational effects [46]. It can be explained as follows:

(1) y=f(t,w,l…)+ε 

where *y* is the structural response (i.e. natural frequency), f(t,w,l…) is the function characterizing the environmental/operational effects (i.e. temperature *t*, wind *w* and traffic load *l*…) and ε is a stationary Gaussian variable that is associated with structural modification and measurement noise. Under normal operational conditions, a slight change of the responses caused by structural modifications ε can often be masked by the significant environmental/operational influences f(t,w,l…). As a result, it is essential to quantify and remove such environmental/operational influences to extract the modification-sensitive features for diagnosing the structural health state.

The correlation analysis performed in Section 4 indicates that nonlinear relations between temperature measurements and frequencies in different model orders. Thus, for every individual model order, the Equation (1) is simplified as

*y* = *f*(*t*) + *ε* (2)

In current research, multiple linear regression (MLR) is used to remove the nonlinear influences of the temperature on the identified frequency influences *f*(*t*). In statistics, polynomial regression model is a form of MLR that quantifies the relation between the dependent variable *y* and the independent variable *x* as an *n*th order polynomial function, assuming the error term ε is an independent stationary random process. The theoretical explanation of such model is a calculus-based formulation of Maclaurin and Taylor series expansions of functions: any suitably well-behaved function of a mathematical variable *x* can be written as an infinite sum of terms involving increasing powers of *x*. It should be noted that the term “linear” in MLR means that the regression function is linear in the independent variable *x* and its coefficients; thus, the polynomial regression model can be used to characterize the nonlinear relationship between frequency and temperature.

Once the *f*(*t*) is identified, the environmental influences can be removed as:

(3) ε=y−f(t) 

Subsequently, the novelty detection technique can be applied on the error term ε to detect early structural changes. An internal representation novelty index (*NI*) is first built when the structure is under healthy conditions covering one full cycle of environmental/operational variations, and then data are subsequently examined to observe the possible occurrence of significant departure from the normal condition by using the outlier analysis [47].

### 6.2. Multiple Linear Regression

The identified frequencies in the each individual model order can be expressed as a polynomial regression function of the temperature measurements as follows:

(4) $$\bar{y}=T\bar{\beta }+\bar{\varepsilon }\;$$

(5) $$\bar{y}=\left[\begin{array}{l} y_{1}\\ \cdots \\ y_{i}\\ \cdots \\ y_{N} \end{array}\right],T=\left[\begin{array}{llllllllll} 1 & t_{1,1} & \cdots & t_{j,1} & (t_{1,1}){}^{2} & \cdots & (t_{j,1}){}^{2} & (t_{1,1}){}^{l} & \cdots & (t_{j,1}){}^{l}\\ \cdots & & & & & & & & & \\ 1 & t_{1,i} & \cdots & t_{j,i} & (t_{1,i}){}^{2} & \cdots & (t_{j,i}){}^{2} & (t_{1,i}){}^{l} & \cdots & (t_{j,i}){}^{l}\\ \cdots & & & & & & & & & \\ 1 & t_{1,N} & \cdots & t_{j,N} & (t_{1,N}){}^{2} & \cdots & (t_{j,N}){}^{2} & (t_{1,N}){}^{l} & \cdots & (t_{j,N}){}^{l} \end{array}\right],\bar{\varepsilon }=\left[\begin{array}{l} \varepsilon_{1}\\ \cdots \\ \varepsilon_{i}\\ \cdots \\ \varepsilon_{N} \end{array}\right]\;$$

where *y_i_* means the identified frequency, *i* is the index of the total *N* samples for regression analysis, *j* represents the number of the input variables, *l* is the order of the polynomial model and *ε* is approximately assumed as a random variable.

The coefficient vector $\bar{\beta }={\left[\beta_{0}\ldots \beta_{j}\ldots \beta_{lj}\right]}^{T}$ can be solved by classical least-square estimator by minimizing the sum of squares of the errors with respect to *β*:

(6) $$\underset{}{\min({\bar{\varepsilon }}^{T}\bar{\varepsilon })=}\underset{}{\min[{\left(\bar{y}-T\bar{\beta }\right)}^{T}(\bar{y}-T\bar{\beta }))}]\;$$

$\bar{\beta }$ can be estimated as

(7) $$\bar{\beta }={\left[T^{T}T\right]}^{-1}T^{T}y\;$$

The residual error vector *ɛ* corresponding to each model order can be given as:

(8) $$\bar{\varepsilon }=\bar{y}-T\bar{\beta }=\bar{y}-T{\left[T^{T}T\right]}^{-1}T^{T}y\;$$

The above equation can also be integrated all model orders as a matrix form:

(9a) E=Y−TB 

where

(9b) $$E=[{\bar{\varepsilon }}^{1}\ldots {\bar{\varepsilon }}^{k}\ldots {\bar{\varepsilon }}^{m}],Y=[{\bar{y}}^{1}\ldots {\bar{y}}^{k}\ldots {\bar{y}}^{m}],B=[{\bar{\beta }}^{1}\ldots {\bar{\beta }}^{k}\ldots {\bar{\beta }}^{m}]\;$$

*k* is the order of the model and *m* represents the number of the model orders. *E*, *Y* and *B* are residual, frequency and coefficient matrix, respectively. 

Various polynomial regression models can be considered depending on the number of the input variables and the corresponding orders. In order to select the optimal model, both coefficient of determination *R*^2^ and Akaike’s Information Criterion (*AIC*) [48] are used. The former, *R*^2^, is defined as:

(10) $$R^{2}=1-SSE/SSTO\;$$

And

(11) $$SSE=\sum_{i=1}^{N}{\left(y_{i}-{\tilde{y}}_{i}\right)}^{2},\;SSTO=\sum_{i=1}^{N}{\left(y_{i}-y_{mean}\right)}^{2}\;$$

where *SSE* and *SSTO* denote the residual sum of squares and total sum of squares, respectively. *y_mean_* refers to the mean of the total *N* samples and ${\tilde{y}}_{i}$ is the *ith* element of the best fit. *R*^2^ explains the percentage of measured data that is closest to the best fit line. The closer it is to 1, the better measured data fit the regression curve. However, the drawback of the coefficient of determination *R*^2^ is that it fails to distinguish a good model and an over-fit model because it always improves with increasing model complexity. In order to trade off goodness-of-fit and complexity of models objectively, the most popular alternative is the Akaike Information Criterion (*AIC*).

(12) AIC=Nln(SSE)+2p 

where *p* is the number of estimated coefficients and *N* is the samples used in regression analysis. Usually, the model that gives the smallest value of *AIC* statistic is the preferred one. Hence, *AIC* not only rewards goodness of fit, but also includes a penalty that is an increasing function of the number of estimated coefficients. For the selected optimal model, both a higher *R^2^* and a smaller *AIC* are expected.

Apart from *R^2^* and *AIC*, examination of the residual vector *ε* also assists to evaluate the goodness of the model. If the *ε* approximately has a normal distribution, it suggests that the selected model accurately characterizes the measured data. Moreover, the residual vector *ε* should be independent with input variables, which indicates that the model appropriately represents the relationships between the dependent variable *y* and the independent variable *x*.

### 6.3. Detection of Structural Modifications

The coefficient matrix *B* can be estimated by applying Equations (7)–(9) using frequency matrix *Y* and temperature matrix *T* within the baseline year. As shown in Equation (9b), substitution of estimated coefficient matrix *B* to frequency matrix *Y* and temperature matrix *T* in the following years leads to the residual matrix $E=[{\bar{\varepsilon }}^{1}\ldots {\bar{\varepsilon }}^{k}\ldots {\bar{\varepsilon }}^{m}]$, which corresponds to the dynamic features from which the environmental and operational effects have been removed. 

Application of the novelty detection technique on the error matrix *E* can be applied to detect early structural changes. Firstly, the novelty index (*NI*) can be defined as its Euclidean norm:

(13) NI=‖E‖ 

Subsequently, to detect possible structural modification, an *X*-bar control chart can be constructed by drawing two lines: a center line (*CL*) and an additional horizontal line corresponding to an upper limit (*UCL*), which are:

(14a) CL=μ 

(14b) UCL=μ+ασ 

where μ and σ are the mean value and standard deviation of *NI* in the reference healthy state. α is taken as 3, corresponding to 99.7% confidence.

Two criteria are employed as structural modification warning: (1) the ratio of the mean values of *NI* between a healthy and a subsequent state; and (2) the percentage of *NI* lying outside the defined limit by outlier analysis. If the structure is still healthy, the new residues in different orders of the subsequent state should stay at the same level as the reference state, so the mean values of *NI* under the healthy/subsequent state should be approximately the similar value, and the percentage of *NI* exceeding the defined upper limit should be small. Conversely, with the occurrence of structural changes, the new residual vectors will depart from the hyper-plane in the reference state, which will result in a relatively large *NI* ratio under healthy/subsequent state and cause the percentage of *NI* outside the limit to increase significantly.

## 7. Application to Continuous Dynamic Measurements of the Westend Bridge

The variations of both temperature measurements and frequencies over 14 years are shown in Figure 3 and Figure 8, respectively. In order to compare the features extracted from strain measurements, the baseline year starts at the last week in November 2000 and ends at the last week in November 2001, covering all operational conditions for a full cycle time. The data in the baseline year is treated by MLR in order to estimate the coefficient vector in each model order. Then, the coefficient vector ${\bar{\beta }}^{k}(k=1,2,\ldots ,5)$ is applied to the data in the following years to detect the structural modifications. It should be noted that the temperature recorded by T1–T5 ranges from −5.3 °C to 44.4 °C. In the following years, if the corresponding temperature falls in this range, the frequencies are selected to calculate the residual matrix ***E***; otherwise, when the temperature is out of this range, the frequencies are discarded because the coefficient vector ${\bar{\beta }}^{k}(k=1,2,\ldots ,5)$ in the baseline year are estimated only considering the temperature range from −5.3 °C to 44.4 °C.

### 7.1. Model Estimation

The selection of polynomial regression model depends on the number of both the input variables and polynomial model orders. In current research, all temperature measurements from 5 different sensors are firstly selected as input variables. Only the model order *l* is determined by the *R^2^* and *AIC*. The frequencies in 5 different modes in the baseline year are separately substituted in Equations (6)–(9). The variable *j* is 5 because all thermal sensors are considered. The polynomial order ranges from 1 to 5 and the corresponding *R^2^* and *AIC* can be calculated according to Equations (10)–(12).

**Figure 11:**
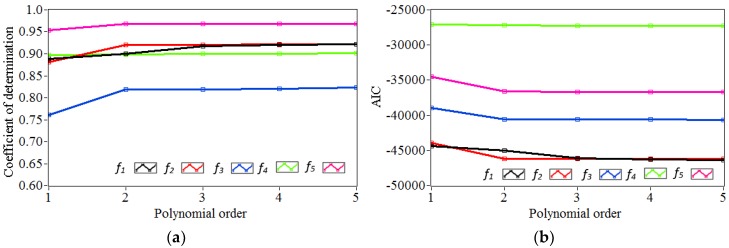
Variation of both *R^2^* and *AIC* corresponding to frequencies *f_1_*–*f_5_* in different modal orders versus increasing polynomial orders *l*: (**a**) *R^2^*; (**b**) *AIC*.

Figure 11 shows both *R^2^* and *AIC* as a function of the polynomial order *l*. The corresponding values are listed in Table 3. It is observed that, for each polynomial order, the *R^2^* increases while the *AIC* decreases with the rising polynomial order. For *f_1_* and *f_2_*, *R^2^* and *AIC* do not change significantly at the 4th order. With regard to *f_3_*, *f_4_* and *f_5_*, *R^2^* and *AIC* are stable at the 3rd order. It is decided that the 4th order polynomial regression model is used to characterize the nonlinear relationships between temperature and frequencies in different model orders. For each model order, the coefficient vector consists of lj + 1 = 4 × 5 + 1 = 21 parameters to be estimated. It is worth noting in Table 3 that *R^2^* ranges from 0.820 (*f_3_*) to 0.968 (*f_5_*). The higher values of *R^2^* indicate a better fit between the measured and simulated frequencies.

### 7.2. Model Validation

Examination of the sequence of residuals is an efficient way to evaluate the quality of the estimated model. If the residuals can be regarded as Gaussian white noise as assumed in Equation (4), it suggests that the model accurately simulates the information of the data. The degree of “whiteness” of the residuals can be assessed by the autocorrelation functions, based on the assumption that the autocorrelation function of a Gaussian white noise is a delta function.

**Figure 12:**
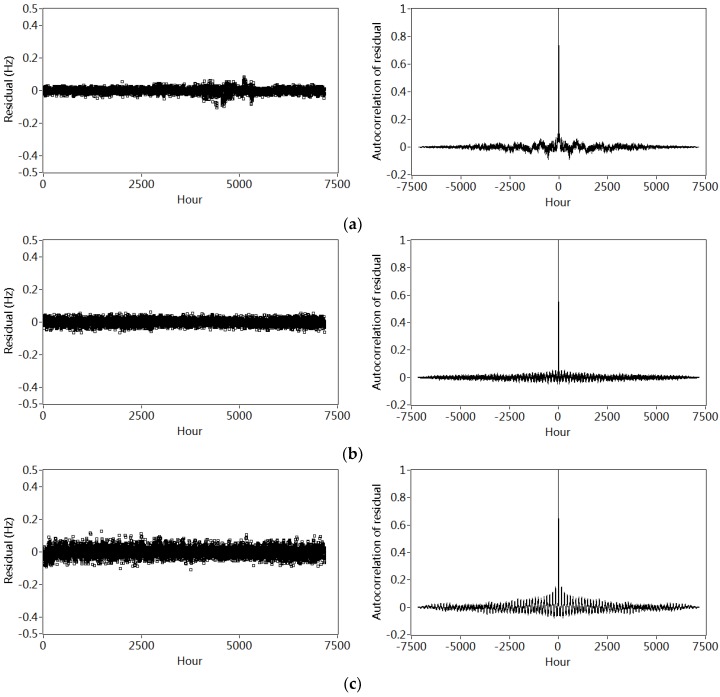

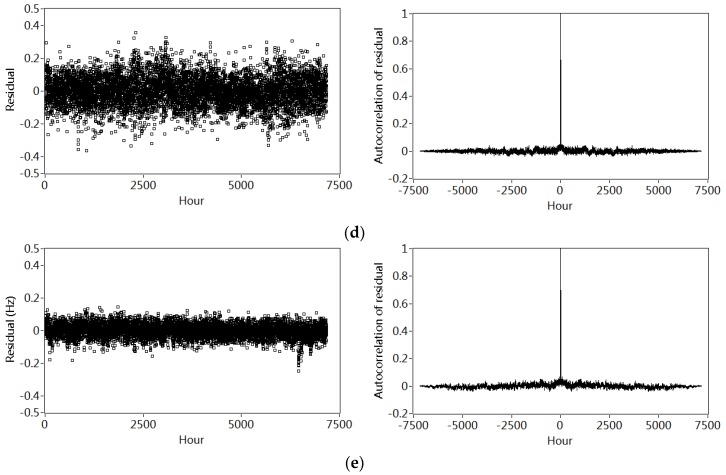
Residuals between the identified and calculated frequencies in different modes (left side) and the corresponding autocorrelation (right side): (**a**) Mode 1; (**b**) Mode 2; (**c**) Mode 3; (**d**) Mode 4;(**e**) Mode 5.

The plots on the left sides of Figure 12a–e show the residuals vectors calculated based on the Equation (9) by applying the polynomial models with four orders to the data in the baseline year 2000/2001. For modes 4, 5, 3, 2 and 1, their residuals decrease gradually, suggesting a better quality of model fits. The autocorrelation functions of these residuals are shown in the right sides. It is noticed that each function displays an obvious spike at zero, with the rest of values close to zero. Inspection of both the residuals and their autocorrelation functions suggests that the proposed 4th order polynomial regression model might account for the nonlinear relationship between the frequencies and temperature variations.

**Figure 13:**
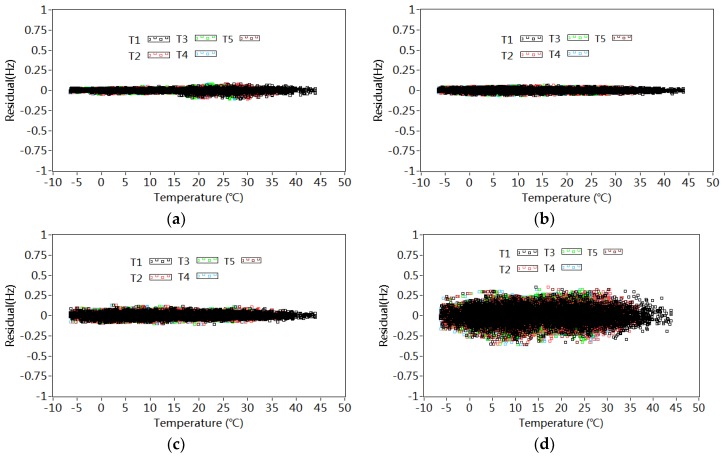

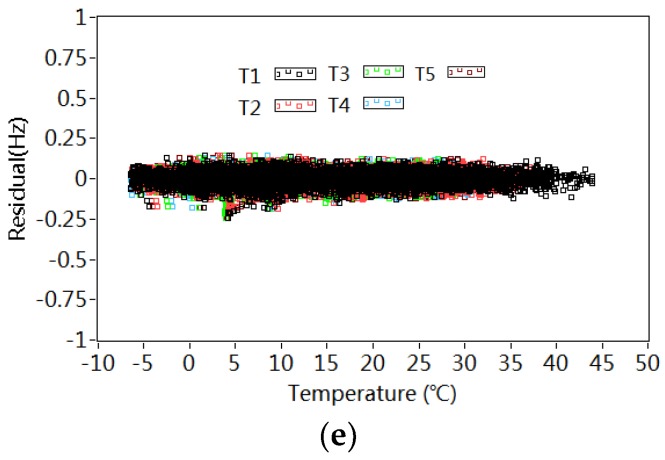
Relationship between the residuals in different modes and temperature T1–T5: (**a**) Mode 1; (**b**) Mode 2; (**c**) Mode 3; (**d**) Mode 4; (**e**) Mode 5.

The quality of the model’s fit can also be further evaluated by the relationship between the residues ${\bar{\varepsilon }}^{1}-{\bar{\varepsilon }}^{5}$ and temperature variations as shown in Figure 13. There are no clear tendencies between the residuals in different modes and temperature variations T1–T5. It may be concluded that nonlinear influences of temperature variations on the frequencies are efficiently removed by the 4th order polynomial regression model. 

### 7.3. Detection of Structural Change over 14 Years

Once the coefficient vector ${\tilde{\beta }}^{i}$ for each model order in the baseline year 2000/2001 is estimated, the residual vector ${\tilde{\varepsilon }}^{i}$ in the following years can be calculated by comparing the measured frequency $y^{i}$ and estimated $t{\tilde{\beta }}^{i}$ according to Equation (8). Then, the novelty index (*NI*) can be computed using Equation (13) for tracking the long-term evolution’ of the bridge from 2000 to 2013.

**Figure 14:**
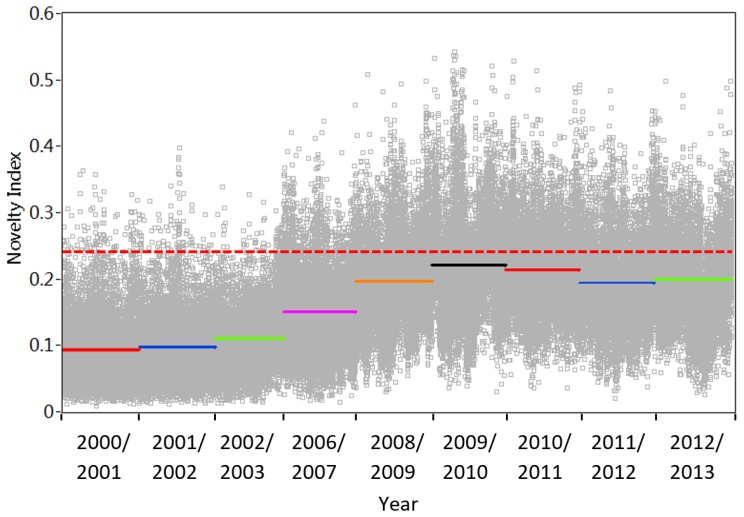
Variation of novelty indices considering five temperature records T1–T5.

**Figure 15:**
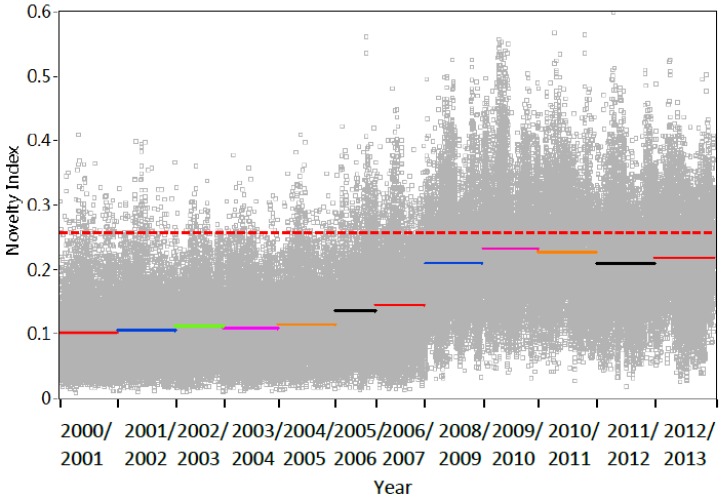
Variation of novelty indices considering four temperature records T1–T4.

It is observed in Figure 3c that T1 failed to work from August 2004 to December 2006 and March 2006 to August 2006. Thus, firstly T1 to T5 are all considered for evaluating the long-term bridge modification, and then only T1 to T4 are substituted in Equations (9), (13) and (14). Figure 14 and Figure 15 show the variations of the novelty indices from the end of 2000 to the end of 2013, considering temperature measurements T1–T5 and T1–T4, respectively. Table 4 lists the results of outlier analysis and ratio of mean *NI* ($\bar{NI}$).

Figure 14 and Figure 15 show the variations of the novelty indices from the end of 2000 to the end of 2013, considering temperature measurements T1–T5 and T1–T4, respectively. Table 4 lists the results of outlier analysis and ratio of mean *NI* ($\bar{N}\bar{I}$). When five temperature inputs are considered, it is noted from Figure 14 that the annual fluctuations due to variation of temperature in years 2000/2001 and 2001/2002 are removed. Table 5 lists the ratio of the mean value of *NI* and outlier analysis results. It can be seen that 1.37% *NI* in year 2001/2002 and 2002/2003 fall out of the range of the upper line and the ratio between the mean values of *NI* in the first two years is only 1.04. These indices suggest that no significant modification occurs on this bridge. By contrast, it is interesting to notice from Figure 14 that the ratio of $\bar{N}\bar{I}$ gradually increases and more and more novelty indices scatter out of the upper limit from year 2000/2001 to 2012/2013. In particular, a clear gap is observed in the novelty indices between 2006/2007 and 2008/2009. The ratio of $\bar{N}\bar{I}$ in Figure 14 reaches the maximum in year 2009/2010 and stay at a relatively stable level. Meanwhile, considering five input variables, inspection of Table 4 shows that from year 2006/2007 to 2007/2008, the ratio of $\bar{N}\bar{I}$ jumps from 1.61 to 2.10, the percentage of outlier analysis increases from 8.66 to 22.28. All of these results reach their peak in 2009/2010.

The similar phenomenon is also observed in Figure 15 as well as the corresponding outlier analysis and ration of $\bar{N}\bar{I}$ in Table 4, when 4 temperature measurements are considered. Such results may suggest that the bridge experiences a structural change during the period from 2000 to 2013.

## 8. Discussion

In order to partially explain the detected structural changes observed in Figure 14 and Figure 15, the finite element model is updated on the basis of the strain measurements.

**Figure 16:**
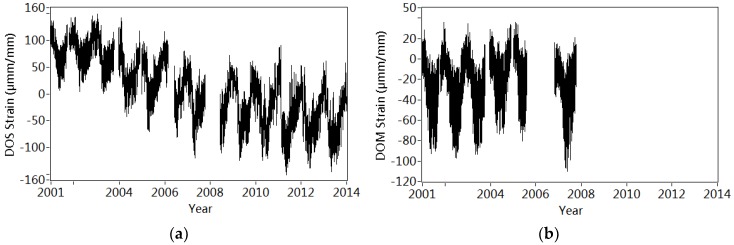

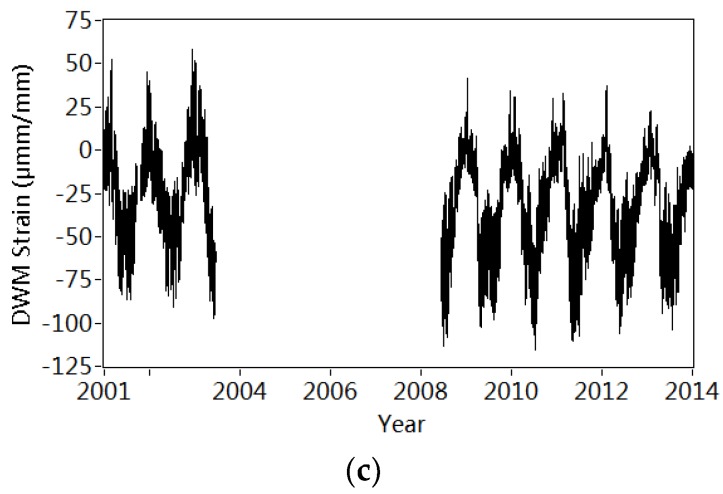
Variation of strain from year 2001 to year 2014: (**a**) Strain measured in the prestressed tendon (DOS); (**b**) Strain measured in the east concrete web (DOM); (**c**) Strain measured in the west concrete web (DWM).

Figure 16 shows the variation of 250 strain measured from the main prestressed tendon in the east web (DOS), strain recorded from both east concrete web (DOM) and the west concrete web (DWM). The positions of DOS, DOM and DWM are shown in Figure 2. Table 5 lists the annual averaged value of DOS, DOM and DWM. It is interesting to notice that the annual strain in the prestressed tendon gradually decreases from 85.29 μmm/mm to −43.99 μmm/mm from 2000 to 2013, suggesting the loss of prestress in the tendons. On the contrary, the concrete strain DWM changes slightly from −23.93 μmm/mm to −31.76 μmm/mm during 14 years [49].

The loss of prestress in the tendon could be approximately simulated by reducing the initial press of the pre-stressed tendons. And the annual loss of the stress is calculated as:

(15) $$\sigma =E\varepsilon_{re}\;$$

where σ are the yearly losing stress of each year (2000–2013), and $\varepsilon_{re}$ represents the loss of annual average strain from 2000 to 2013. *E* is the elastic modulus of the prestressed tendon.

**Figure 17:**
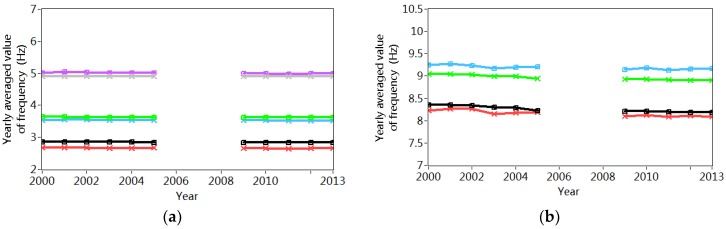
The variation of calculated and estimated frequencies from 2001 to 2013: (**a**) 1st–3rd mode; (**b**) 4th-5th mode.

Subsequently, the updated finite model approximately considering the loss of prestress is used to simulate the annual reduction of frequencies. Figure 17 shows both the calculated and estimated annual mean values of frequencies. Similar decreasing tendencies are observed in both calculated and estimated frequencies, which may partially demonstrate that the loss of prestress leads to the structural changes reflected by the deviation of $\bar{N}\bar{I}$ during 14 years.

In [49], another statistical health index is extracted from the relationship between stains in both concrete and prestressed tendon. The tendency of the strain-based health index also suggests the long-time bridge change, which may be partially validated the observations on the basis of the frequency-based health index in current paper.

It is also interesting to note in [50] that the Berlin Senate Department for Urban Development declared that the Westend Bridge was blocked to “heavy duty and heavy load traffic”. Although this bridge is to be replaced with a new one, there is no clear timetable, which “causes the highways to no longer justify their intended purpose”. Such descriptions indicate the existence of possible deterioration in the aging Westend Bridge built in 1965.

## 9. Conclusions

This paper describes the detection of structural change of a prestressed concrete box-beam highway bridge using the continuous dynamic monitoring data acquired from 2000 to 2013 in the context of statistical pattern recognition. After rehabilitation, the Westend bridge was implemented with an integrated health monitoring system in order to alarm the potential changes under normal operational conditions. Five thermal sensors deployed in a section characterize the spatial distribution of temperature. The traffic density is approximately estimated by the dynamic strain measured in the prestressed tendon. The frequencies in five model orders are estimated by the structural vibrations recorded by 20 velocity sensors. 

During 14 years, both the highest (49.3 °C) and lowest temperature (−14.6 °C) are captured by the thermal sensor in the asphalt. Annual frequency variation ratios of the five modes change from 7.4% to 17.5%. It is observed that the temperature is the driving reason for the variations of frequencies, and the averaged values of frequencies in five orders gradually decrease in the last 14 years. The 4-order polynomial regression function is used to remove the temperature effect, and corresponding residues are defined as features and treated by statistical treatment. The extracted health indices consists of both outlier analysis of *NI* and the ratio of mean values of *NI*. If five thermal measurements are considered, the outlier analysis of *NI* increase significantly from 1.37% in year 2000/2001 to over 21.59% after 2008. Similarly, the ratio of mean values of *NI* increases gradually from 1.00 in 2000/2001 to over 2.06 after 2008. Obvious deviation of the proposed health indices may suggest the structural change during 14 years. Meanwhile, the annual averaged dynamic strain measured in the main prestressed tendon also progressively decrease from 85.29 μmm/mm to −43.99 μmm/mm from 2000 to 2013. Similar decreasing tendencies are observed in both calculated and monitored annual mean values of frequencies, according to numerical simulation considering the progressively loss of prestress in the tendons. The research presented in the current paper partially proves that the vibration-based SHM system can capture the gradually realistic deterioration for aging infrastructures over wide time scales under the framework of statistical pattern recognition.

In the future, the mechanism of temperature effect on frequencies will be further investigated considering the variation of strain, inclination, and cracks.

## Figures and Tables

**Table 1:** Annual variation of the frequencies corresponding to the bending mode.

Year	Mode 1			Mode 2			Mode 4		
	Annual Averaged Value (Hz)	Yearly Variation Range (Hz)	Yearly Relative Variation Ratio (%)	Annual Averaged Value (Hz)	Yearly Variation Range (Hz)	Yearly Relative Variation Ratio (%))	Annual Averaged Value (Hz)	Yearly Variation Range (Hz)	Yearly Relative Variation Ratio (%)
2000	2.68	2.53–2.81	10.5	3.54	3.40–3.73	9.3	8.22	7.50–8.90	17.0
2001	2.68	2.50–2.81	11.6	3.55	3.43–3.72	8.2	8.27	7.53–8.91	16.7
2002	2.68	2.47–2.84	13.8	3.55	3.41–3.75	9.6	8.27	7.57–8.93	16.4
2003	2.65	2.47–2.87	15.4	3.53	3.38–3.74	10.2	8.15	7.53–8.96	17.5
2004	2.66	2.50–2.81	11.2	3.53	3.41–3.72	8.8	8.18	7.61–8.87	15.4
2005	2.67	2.47–2.81	12.7	3.54	3.38–3.72	9.6	8.19	7.55–8.95	17.1
2006 2008	/	/	/	/	/	/	/	/	/
2009	2.65	2.47–2.87	15.1	3.53	3.34–3.75	11.6	8.10	7.53–8.89	16.8
2010	2.65	2.47–2.84	14.0	3.53	3.31–3.75	12.5	8.13	7.40–8.80	17.2
2011	2.64	2.45–2.81	13.6	3.51	3.32–3.70	10.8	8.08	7.47–8.75	15.8
2012	2.65	2.47–2.91	16.6	3.52	3.34–3.76	11.9	8.11	7.50–8.92	17.5
2013	2.66	2.44–2.81	13.9	3.52	3.36–3.70	9.7	8.09	7.43–8.76	16.4

**Table 2:** Annual variation of the frequencies corresponding to the bending mode.

Year	Mode 3			Mode 5		
	Annual Averaged Value (Hz)	Yearly Variation Range (Hz)	Yearly Relative Variation Ratio (%)	Annual Averaged Value (Hz)	Yearly Variation Range (Hz)	Yearly Relative Variation Ratio (%)
2000	5.02	4.85–5.26	8.2	9.25	8.75–9.87	12.1
2001	5.04	4.88–5.26	7.5	9.27	8.81–9.80	10.7
2002	5.03	4.87–5.27	8.0	9.24	8.68–9.86	12.8
2003	5.01	4.85–5.27	8.4	9.17	8.71–9.83	12.2
2004	5.02	4.87–5.26	7.8	9.20	8.79–9.78	10.8
2005	5.02	4.85–5.26	8.2	9.21	8.77–9.72	10.3
2006 2008	/	/	/	/	/	/
2009	4.99	4.83–5.27	8.8	9.15	8.74–9.88	12.5
2010	4.99	4.84–5.27	8.6	9.18	8.71–9.95	13.5
2011	4.98	4.80–5.20	8.0	9.13	8.74–9.71	10.6
2012	4.99	4.84–5.30	9.2	9.16	8.72–9.87	12.6
2013	4.99	4.84–5.22	7.6	9.17	8.72–9.73	11.0

**Table 3:** Values of both *R^2^* and *AIC* corresponding to frequencies in different modal orders versus increasing polynomial orders.

Modal Order		Polynomial Order				
		1	2	3	4	5
*f_1_*	*R^2^*	0.888	0.899	0.917	0.920	0.921
	*AIC*	−44410	−45032	−46097	−46323	−46400
*f_2_*	*R^2^*	0.881	0.920	0.920	0.921	0.921
	*AIC*	−43930	−46151	−46181	−46224	−46230
*f_3_*	*R^2^*	0.760	0.818	0.819	0.820	0.821
	*AIC*	−38981	−40556	−40571	−40586	−40689
*f_4_*	*R^2^*	0.897	0.898	0.900	0.900	0.901
	*AIC*	−27071	−27140	−27225	−27227	−27230
*f_5_*	*R^2^*	0.953	0.967	0.968	0.968	0.968
	*AIC*	−34527	−36575	−36659	−36670	−36678

**Table 4:** Outlier analysis and ratio of mean of *NI* ($\bar{N}\bar{I}$) considering both 4 and 5 temperature measurements in different years.

Year	Outlier Analysis (%)		Ratio of $\bar{NI}\;$	
	4 temp	5 temp	4 temp	5 temp
2000/2001	1.34	1.37	1.00	1.00
2001/2002	1.40	1.37	1.04	1.04
2002/2003	0.93	1.22	1.09	1.17
2003/2004	0.94	/	1.07	/
2004/2005	1.53	/	1.13	/
2005/2006	5.86	/	1.34	/
2006/2007	5.98	8.66	1.42	1.61
2007/2008	/	/	/	/
2008/2009	23.65	22.28	2.06	2.10
2009/2010	33.88	36.45	2.29	2.35
2010/2011	32.77	30.93	2.23	2.27
2011/2012	24.81	21.59	2.06	2.06
2012/2013	29.23	24.44	2.14	2.12

**Table 5:** Annual variation of strains measured in both prestressed tendon and concrete webs.

Year	Strain in Prestressed Tendon (DOS)		Strain in East Concrete Web (DOM)		Strain in West Concrete Web (DWM)	
	Averaged Value (μmm/mm)	Range (μmm/mm)	Averaged Value (μmm/mm)	Range (μmm/mm)	Averaged Value (μmm/mm)	Range (μmm/mm)
2000	/	/	/	/	/	/
2001	85.29	6.94–140.98	−20.47	−92.50–35.54	−23.93	−86.43–52.72
2002	86.23	4.50–144.59	−22.79	−97.18–29.55	−23.85	−90.84–58.06
2003	70.40	1.02–148.01	−26.49	−93.62–34.25	/	/
2004	40.49	−41.94–141.38	−11.16	−73.40–34.23	/	/
2005	32.50	−70.30–115.29	−13.33	−80.74–35.81	/	/
2006	/	/	/	/	/	/
2007	−11.21	−119.19–71.44	−28.88	−110.39–20.75	/	/
2008	/	/	/	/	/	/
2009	−19.91	−123.58–60.72	/	/	−33.23	−102.67–41.84
2010	−24.70	−123.62–58.01	/	/	−31.27	−115.60–30.87
2011	−38.08	−150.79–89.72	/	/	−33.22	−110.78–32.80
2012	−43.36	−137.60–57.24	/	/	−33.63	−106.05–37.48
2013	−43.99	−144.95–61.28	/	/	−31.76	−103.88–22.41
